# Causes of death among patients with cutaneous melanoma: a US population-based study

**DOI:** 10.1038/s41598-023-37333-4

**Published:** 2023-06-24

**Authors:** Mohammed Ahmed Sadeq, Mohamed Hady Ashry, Reem Mohammed Farouk Ghorab, Abdelrahman Yousry Afify

**Affiliations:** 1 Faculty of Medicine, Misr University for Science and Technology, 6th of October, Giza, Egypt; 2 School of Medicine, New Giza University (NGU), Giza, Egypt

**Keywords:** Cancer, Melanoma

## Abstract

Research on mortality outcomes and non-cancer-related causes of death in patients with cutaneous melanoma (CM) remains limited. This study aimed to identify the prevalence of non-cancer-related deaths following CM diagnosis. The data of 224,624 patients diagnosed with malignant CM in the United States between 2000 and 2019 were extracted from the Surveillance, Epidemiology, and End Results (SEER) database. We stratified our cohort based on their melanoma stage at diagnosis and further calculated standardized mortality ratios (SMRs) for each cause of death, comparing their relative risk to that of the general US population. The total number of fatalities among melanoma patients was 60,110, representing 26.8% of the total cases. The percentage of deaths is directly proportional to the disease stage, reaching 80% in distant melanoma. The highest fatalities among the localized melanoma group (25,332; 60.5%) occurred from non-cancer causes, followed by melanoma-attributable deaths (10,817; 25.8%). Conversely, melanoma is the leading cause of death in regional and distant melanoma cohorts. Cardiovascular and cerebrovascular diseases were the most prevalent non-cancer causes of death among the three disease-stage cohorts. Compared to the general population, we did not observe an increased risk of death due to non-cancer causes in the localized CM cohort, while patients diagnosed with regional and distant CMs had a statistically significant higher risk of death from all the reported major causes of death.

## Introduction

Melanoma is an aggressive type of cancer that usually originates in the skin, typically starting out as a mole following cell DNA damage related to ultraviolet (UV) radiation exposure from the sun or tan beds, with an increased risk in old, light-skinned, positive family history, and immunosuppressed individuals. It often arises from cutaneous epidermal melanocytes; however, it can occur in any melanocyte-containing tissue, such as the genitourinary and uveal tracts^1^. Although melanoma only makes for less than 10% of skin cancer cases^2^, it remains the deadliest type owing to its aggressiveness and high mortality rate. In addition, the incidence of melanoma is increasing at an alarming rate faster than any other solid tumor, becoming the fifth most common cancer in the United States^1^.

The pathophysiology of melanoma involves the uncontrolled growth and spread of abnormal melanocytes. These cells can arise from existing moles or develop in previously healthy skin. Melanoma can be classified into four stages, with stage I being the earliest and stage IV the most advanced. In the early stages, cancer is confined to the outermost layer of the skin, but as it progresses, it can invade deeper layers of the skin and spread to other parts of the body via the lymphatic system or bloodstream^3^.

The stage of the disease, the site of growth, and the patient’s general health all influence melanoma treatment. The main therapy for early-stage melanoma is surgical excision, which includes the removal of the cancerous lesion as well as a margin of nearby healthy tissue. In some instances, lymph nodes may be removed to avoid the spread of cancer. In contrast, chemotherapy, radiation therapy, immunotherapy, and targeted therapy are therapeutic options for advanced-stage melanoma. Despite the availability of successful therapies, melanoma remains a major public health concern, especially in areas with high UV radiation exposure. Early detection and therapy are critical for better outcomes in melanoma patients^4^.

Compared with other cancers, patients with melanoma have a higher risk of dying from non-cancer causes than from melanoma itself^5^. In a recent prospective observational study, the causes of death in cancer patients, especially those receiving chemotherapy, were analyzed. In addition to cancer progression, other causes of death such as thromboembolism (both arterial and venous), infection, respiratory failure, bleeding, and aspiration pneumonitis have been previously reported^6^.

Although determining the causes of death in cancer patients is important to evaluate treatment efficacy and complications, as well as to construct an effective health surveillance policy in cancer survivors, the literature on the causes of death in patients with cutaneous melanoma remains scarce. In the current study, we aimed to utilize the Surveillance, Epidemiology, and End Results (SEER) database to investigate the causes of mortality in cutaneous melanoma patients and compare the risk of death from each reported cause to the general US population.

## Methods

We conducted a retrospective observational cohort study in accordance with the Strengthening the Reporting of Observational Studies in Epidemiology (STROBE) guidelines. The Surveillance, Epidemiology, and End Results (SEER) program was used in this study. Using the SEER*Stat software, we extracted the data of invasive melanoma patients with histological confirmation from 2000 to 2019 from the SEER 17 registries which cover approximately (26.5%) of the general US population. Cutaneous melanoma patients were identified using the variable “melanoma of the skin” in the recode “Site Recode ICD-O-3/WHO 2008” which includes the histological codes (8720-8790) and site codes (C440-C449). All patients were followed until the time of recorded death or at the end of the study period. Institutional review board approval was not required for this study because the SEER data is anonymized and considered non-human research.

Given the differences in the nature of the disease and treatment protocols, we hypothesized that the stage of melanoma would have a great impact on the cause of death. We used the SEER summary stage variable as the main discriminating variable in this study as it is the most complete and consistent staging variable in the SEER 17 database, covering the greatest number of patients and years of diagnosis. Accordingly, invasive melanoma was classified into three distinct entities: localized melanoma, which encompasses Clark levels II-IV without lymph node involvement; regional melanoma, which encompasses Clark level V and/or any regional lymph node involvement regardless of the direct extension level; and distant melanoma, which encompasses distant lymph nodes and organ metastasis. All patients with unknown disease stage were excluded from the current analysis.

Errors in reporting the causes of death may occur in large databases. For example, melanoma-attributable death caused by distant organ metastasis can be falsely classified as distinct secondary cancer-attributable death. To eliminate the risk of misclassification, we used the “SEER cause-specific death classification” variable to identify melanoma-attributable deaths as well as the “SEER other cause of death classifications” variable to better identify non-cancer CODs. These variables take into consideration multiple factors, such as patient comorbidities and the total number of recorded malignancies for each patient and are therefore considered more accurate. We further stratified the CODs into four different periods: deaths in the first year after diagnosis, 1–5 years, 5–10 years, and more than 10 years after melanoma diagnosis. The CODs were determined based on the ICD-10-WHO classification.

Standardized mortality ratios (SMRs) were calculated for each cause of death in our cohort. SMRs were defined as the observed-to-expected ratio (O/E), where “observed” represents the number of patients with melanoma who died from any defined cause of death within a specific timeframe, and “expected” represents the number of people who are expected to die from the same cause of death in a demographically similar general population within the same period. General mortality data for the US population were collected by the National Center for Health Statistics. Using the SEER*Stat software (version 8.4.0.1) we estimated SMRs with 95% confidence intervals (CIs). All statistical tests were two-sided, and a *p*-value of less than 0.05 was considered statistically significant.

## Results

### Baseline characteristics

Of the included 224,624 patients analyzed in our study, the majority were diagnosed with localized disease (n = 194,075; 86.4%), while only 21,079 (9.38%) patients were diagnosed with a regional disease, and 9470 (4.21%) patients had confirmed distant disease (Table 1). Males (n = 126,538; 56.33%) were slightly more affected than females (98,086; 43.67%) among different melanoma stages groups. Furthermore, almost all patients in our cohort were white (n = 221,452; 98.6%), and most of them were older than 64 years of age (n = 85,060; 37.87%). The most affected sites by cutaneous melanoma were the trunk (n = 75,054), followed by the upper limbs and shoulders (n = 56,736), lower limbs and hips (n = 41,536), and face/ear (n = 35,591). However, almost half of the mortalities recorded (n = 17,089) occurred in patients suffering from cutaneous melanoma in the face/ear, followed by the scalp/neck (n = 6033; 35.3%). The baseline characteristics of each group are presented in Table 1.

**Table 1:** Baseline characteristics of all patients with melanoma and number of deaths according to the time of death following diagnosis.

Stage	Group	Patients, No	Timing of deaths after diagnosis, No. (%)				
			All Years	< 1 years	1 to < 5 years	5 to < 10 years	≥ 10 years
All	Stage						
	Localized	194,075	41,859 (100)	3057 (7.3)	17,571 (42)	13,075 (31.2)	8156 (19.5)
	Regional	21,079	10,637 (100)	1697 (16)	6568 (61.7)	1732 (16.3)	640 (6)
	Distant	9470	7614 (100)	5043 (66.2)	2262 (29.7)	241 (3.2)	68 (0.9)
Localized	Age, years						
	< 50	56,973	3108 (100)	100 (3.2)	1211 (39)	1060 (34.1)	737 (23.7)
	50–64	64,932	7804 (100)	344 (4.4)	2937 (37.6)	2505 (32.1)	2018 (25.9)
	> 64	72,170	30,947 (100)	2613 (8.4)	13,423 (43.4)	9510 (30.7)	5401 (17.5)
	Sex						
	Male	106,865	27,606 (100)	2062 (7.5)	11,892 (43.1)	8599 (31.1)	5053 (18.3)
	Female	87,210	14,253 (100)	995 (7)	5,679 (39.8)	4476 (31.4)	3103 (21.8)
	Race						
	White	191,940	41,353 (100)	3016 (7.3)	17,331 (41.9)	12,924 (31.3)	8,082 (19.5)
	Black	621	200 (100)	15 (7.5)	101 (50.5)	61 (30.5)	23 (11.5)
	API	1104	231 (100)	21 (9.1)	108 (46.8)	69 (29.9)	33 (14.3)
	AIAN	410	75 (100)	5 (6.7)	31 (41.3)	21 (28)	18 (24)
	Primary site						
	Face/ear	23,963	8302 (100)	728 (8.8)	3651 (44)	2536 (30.5)	1387 (16.7)
	Scalp and neck	14,049	4153 (100)	376 (9.1)	1942 (46.8)	1222 (29.4)	613 (14.8)
	Trunk	67,410	12,847 (100)	831 (6.5)	5207 (40.5)	4086 (31.8)	2723 (21.2)
	Upper limb and shoulder	51,911	11,084 (100)	766 (6.9)	4508 (40.7)	3554 (32.1)	2256 (20.4)
	Lower limb and hip	36,000	5276 (100)	330 (6.3)	2171 (41.1)	1627 (30.8)	1148 (21.8)
	Other Sites	742	197 (100)	26 (13.2)	92 (46.7)	50 (25.4)	29 (14.7)
	Treatment						
	Surgery	187,913	40,360 (100)	2730 (6.8)	16,900 (41.9)	12,722 (31.5)	8008 (19.8)
	Radiation	503	271 (100)	32 (11.8)	164 (60.5)	62 (22.9)	13 (4.8)
	Chemotherapy	608	358 (100)	66 (18.4)	205 (57.3)	63 (17.6)	24 (6.7)
Regional	Age, years						
	< 50	5708	1881 (100)	200 (10.6)	1212 (64.4)	352 (18.7)	117 (6.2)
	50–64	6735	2926 (100)	387 (13.2)	1895 (64.8)	471 (16.1)	173 (5.9)
	> 64	8636	5830 (100)	1110 (19)	3461 (59.4)	909 (15.6)	350 (6)
	Sex						
	Male	13,188	7,039 (100)	1153 (16.4)	4409 (62.6)	1099 (15.6)	378 (5.4)
	Female	7891	3,598 (100)	544 (15.1)	2159 (60)	633 (17.6)	262 (7.3)
	Race						
	White	20,394	10,257 (100)	1651 (16.1)	6302 (61.4)	1683 (16.4)	621 (6.1)
	Black	264	165 (100)	22 (13.3)	116 (70.3)	18 (10.9)	9 (5.5)
	API	329	166 (100)	17 (10.2)	118 (71.1)	24 (14.5)	7 (4.2)
	AIAN	92	49 (100)	7 (14.3)	32 (65.3)	7 (14.3)	3 (6.1)
	Primary site						
	Face/ear	2158	1173 (100)	186 (15.9)	708 (60.4)	211 (18)	68 (5.8)
	Scalp and neck	2505	1460 (100)	236 (16.2)	934 (64)	210 (14.4)	80 (5.5)
	Trunk	6251	3116 (100)	496 (15.9)	1948 (62.5)	477 (15.3)	195 (6.3)
	Upper limb and shoulder	4125	2030 (100)	308 (15.2)	1227 (60.4)	367 (18.1)	128 (6.3)
	Lower limb and hip	4675	2215 (100)	282 (12.7)	1408 (63.6)	389 (17.6)	136 (6.1)
	Other sites	1365	643 (100)	189 (29.4)	343 (53.3)	78 (12.1)	33 (5.1)
	Treatment						
	Surgery	19,671	9957 (100)	1445 (14.5)	6227 (62.5)	1667 (16.7)	618 (6.2)
	Radiation	1650	951 (100)	194 (20.4)	597 (62.8)	121 (12.7)	39 (4.1)
	Chemotherapy	1676	1029 (100)	232 (22.5)	652 (63.4)	108 (10.5)	37 (3.6)
Distant	Age, years						
	< 50	1868	1360 (100)	873 (64.2)	441 (32.4)	38 (2.8)	8 (0.6)
	50–64	3348	2587 (100)	1714 (66.3)	771 (29.8)	83 (3.2)	19 (0.7)
	> 64	4254	3667 (100)	2456 (67)	1050 (28.6)	120 (3.3)	41 (1.1)
	Sex						
	Male	6485	5276 (100)	3524 (66.8)	1544 (29.3)	155 (2.9)	53 (1)
	Female	2985	2338 (100)	1519 (65)	718 (30.7)	86 (3.7)	15 (0.6)
	Race						
	White	9118	7342 (100)	4881 (66.5)	2165 (29.5)	230 (3.1)	66 (0.9)
	Black	153	115 (100)	71 (61.7)	37 (32.2)	6 (5.2)	1 (0.9)
	API	174	138 (100)	85 (61.6)	50 (36.2)	3 (2.2)	0 (0)
	AIAN	25	19 (100)	6 (31.6)	10 (52.6)	2 (10.5)	1 (5.3)
	Primary site						
	Face/ear	9470	7614 (100)	5043 (66.2)	2262 (29.7)	241 (3.2)	68 (0.9)
	Scalp and neck	539	420 (100)	241 (57.4)	157 (37.4)	14 (3.3)	8 (1.9)
	Trunk	1393	1092 (100)	697 (63.8)	346 (31.7)	41 (3.8)	8 (0.7)
	Upper limb and shoulder	700	518 (100)	297 (57.3)	192 (37.1)	25 (4.8)	4 (0.8)
	Lower limb and hip	861	648 (100)	314 (48.5)	303 (46.8)	29 (4.5)	2 (0.3)
	Other sites	5543	4599 (100)	3329 (72.4)	1117 (24.3)	118 (2.6)	35 (0.8)
	Treatment						
	Surgery	3624	2716 (100)	1395 (51.4)	1145 (42.2)	129 (4.7)	47 (1.7)
	Radiation	2540	2184 (100)	1415 (64.8)	710 (32.5)	49 (2.2)	10 (0.5)
	Chemotherapy	3188	2762 (100)	2008 (72.7)	683 (24.7)	56 (2)	15 (0.5)

### Causes of death for localized cutaneous melanoma

**Figure 1:**
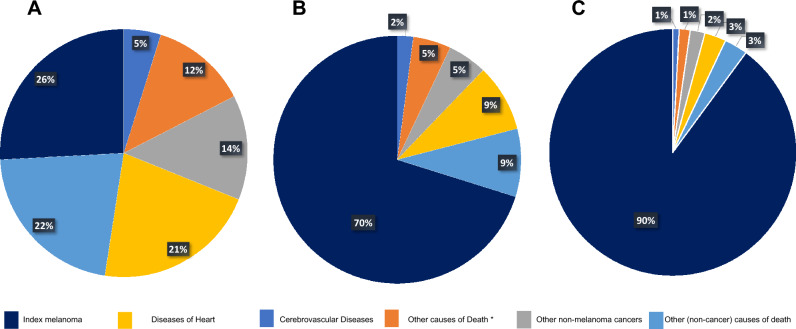
An illustration of causes of death following melanoma diagnosis. (**A**–**C**) Approximated total percentages of deaths across the entire follow-up period for localized, regional, and distant melanoma, respectively. *Deaths coded in the SEER database as “other causes of death”.

The risk of death among localized melanoma patients was lower than the general population within the first year of diagnosis (SMR = 0.74; 95% CI, 0.72–0.77), which was followed by an increasing trend in during the follow-up period. Most deaths in localized disease occurred 10 years after diagnosis (n = 21,231 [50.72%]; SMR = 1.01; 95% CI, 1–1.02), followed by deaths after 1–5 years (n = 9399 [22.45%]; SMR = 1.18; 95% CI, 1.16–1.2) (Table2). In this group, melanoma itself accounted for 10,817 (25.84%) of the deaths, which mostly occurred in the 1–5 years follow-up period (n = 6159; 14.71). Other non-melanoma malignancies accounted for 5710 (13.64%) deaths. Furthermore, the majority of deaths in this group were attributed to non-cancer causes (n = 25,332; 60.52%). The most frequent non-cancer cause of death was diseases of heart accounting for 8939 deaths (35.29% of non-cancer deaths). Other common causes of death among patients with localized cutaneous melanoma were cerebrovascular disease (n = 2080), Alzheimer’s disease (n = 1669), chronic obstructive pulmonary disease (n = 1542), and accidents/adverse events (n = 982). Interestingly, despite the prevalence of non-cancer CODs in this group, we did not observe an increased risk of death compared to the general population, except for deaths attributable to Alzheimer’s disease (n = 1669; SMR, 1.07; 95% CI, 1.02–1.12), and complications of pregnancy and childbirth (n = 10; SMR, 4.97; 95% CI, 2.39–9.15) (Table 2, Fig. 1).

**Table 2:** Standardized mortality ratios for each cause of death following localized melanoma diagnosis.

Cause of death	Timing of deaths after diagnosis									
	< 1 year		1 to < 5 years		5 to < 10 years		≥ 10 years		Total	
	Observed	SMR (95% CI)	Observed	SMR (95% CI)	Observed	SMR (95% CI)	Observed	SMR (95% CI)	Observed	SMR (95% CI)
All	3057	0.74 (0.72–0.77)^b^	9399	1.18 (1.16–1.2)^b^	8172	1.18 (1.16–1.21)^b^	21,231	1.01 (1–1.02)	41,859	1.05 (1.04–1.06)^b^
Melanoma	699	1.82 (1.69–1.96)^b^	6159	6.43 (6.27–6.59)^b^	2939	7.18 (6.92–7.44)^b^	1020	7.36 (6.92–7.83)^b^	10,817	5.72 (5.62–5.83)^b^
Non-melanoma cancers	227	0.26 (0.23–0.29)^b^	2037	0.64 (0.61–0.66)^b^	2027	0.75 (0.72–0.79)^b^	1419	0.77 (0.73–0.82)^b^	5710	0.66 (0.65–0.68)^b^
Non-cancer	2131	0.76 (0.72–0.79)^b^	9375	0.88 (0.87–0.9)^b^	8109	0.88 (0.86–0.9)^b^	5717	0.86 (0.84–0.89)^b^	25,332	0.87 (0.86–0.88)^b^
In situ, benign or unknown behavior neoplasm	1	0.04 (0–0.23)^b^	49	0.54 (0.4–0.71)^b^	61	0.77 (0.59–0.98)^b^	48	0.86 (0.63–1.14)	159	0.63 (0.54–0.74)^b^
Tuberculosis	0	0 (0–5.86)	1	0.46 (0.01–2.55)	2	1.16 (0.14–4.2)	0	0 (0–3.24)	3	0.53 (0.11–1.55)
Septicemia	37	0.73 (0.51–1.01)	128	0.67 (0.56–0.8)^b^	125	0.75 (0.63–0.9)^b^	73	0.62 (0.48–0.78)^b^	363	0.69 (0.62–0.77)^b^
Other infectious and parasitic diseases including HIV	24	0.77 (0.5–1.15)	94	0.83 (0.67–1.01)	77	0.81 (0.64–1.01)	52	0.85 (0.63–1.11)	247	0.82 (0.72–0.93)^b^
Diabetes mellitus	90	0.87 (0.7–1.06)	353	0.91 (0.82–1.01)	276	0.85 (0.75–0.95)^b^	166	0.72 (0.61–0.84)^b^	885	0.84 (0.79–0.9)^b^
Alzheimer’s (ICD-9 and 10 only)	84	0.65 (0.52–0.81)^b^	529	1 (0.92–1.09)	560	1.11 (1.02–1.21)^b^	496	1.23 (1.12–1.34)^b^	1669	1.07 (1.02–1.12)^b^
Diseases of heart	850	0.86 (0.8–0.92)^b^	3381	0.93 (0.9–0.96)^b^	2832	0.93 (0.89–0.96)^b^	1876	0.87 (0.83–0.91)^b^	8939	0.91 (0.89–0.93)^b^
Hypertension without heart disease	31	0.86 (0.58–1.22)	161	1.12 (0.96–1.31)	141	1.07 (0.9–1.26)	89	0.89 (0.71–1.09)	422	1.03 (0.93–1.13)
Cerebrovascular diseases	175	0.86 (0.74–1)^b^	790	1.06 (0.99–1.14)	609	0.96 (0.89–1.04)	454	0.99 (0.9–1.09)	2028	0.99 (0.95–1.04)
Atherosclerosis	10	0.75 (0.36–1.38)	47	1.07 (0.79–1.42)	36	1.1 (0.77–1.52)	30	1.5 (1.01–2.14)^b^	123	1.12 (0.93–1.33)
Aortic aneurysm and dissection	17	0.88 (0.51–1.41)	50	0.76 (0.56–1)	34	0.67 (0.47–0.94)^b^	25	0.78 (0.5–1.15)	126	0.75 (0.63–0.89)^b^
Other diseases of arteries, arterioles, capillaries	12	0.85 (0.44–1.49)	39	0.75 (0.54–1.03)	36	0.82 (0.57–1.14)	23	0.74 (0.47–1.11)	110	0.78 (0.64–0.94)^b^
Pneumonia and influenza	74	0.84 (0.66–1.05)	288	0.9 (0.8–1.01)	228	0.85 (0.75–0.97)^b^	176	0.95 (0.82–1.11)	766	0.89 (0.83–0.95)^b^
Chronic obstructive pulmonary disease and allied conditions	136	0.58 (0.49–0.68)^b^	579	0.65 (0.6–0.71)^b^	486	0.63 (0.57–0.69)^b^	341	0.62 (0.56–0.69)^b^	1542	0.63 (0.6–0.66)^b^
Stomach and duodenal ulcers	7	1.41 (0.57–2.9)	14	0.79 (0.43–1.33)	5	0.35 (0.11–0.81)^b^	14	1.39 (0.76–2.32)	40	0.85 (0.61–1.15)
Chronic liver disease and cirrhosis	20	0.46 (0.28–0.71)^b^	91	0.57 (0.46–0.7)^b^	75	0.56 (0.44–0.7)^b^	61	0.64 (0.49–0.83)^b^	247	0.57 (0.5–0.65)^b^
Nephritis, nephrotic syndrome and nephrosis	46	0.67 (0.49–0.89)^b^	231	0.89 (0.77–1.01)	181	0.8 (0.68–0.92)^b^	117	0.74 (0.61–0.89)^b^	575	0.8 (0.74–0.87)^b^
Complications of pregnancy, childbirth, puerperium	0	0 (0–15.88)	6	7.44 (2.73–16.19)^b^	3	4.73 (0.98–13.82)	1	2.97 (0.08–16.54)	10	4.97 (2.39–9.15)^b^
Congenital anomalies	3	0.75 (0.15–2.18)	15	1.04 (0.58–1.72)	10	0.85 (0.41–1.56)	9	1.1 (0.5–2.09)	37	0.96 (0.68–1.33)
Certain conditions originating in perinatal period	0	0 (0–96.23)	1	11.18 (0.28–62.29)	0	0 (0–66.84)	0	0 (0–102.45)	1	4.57 (0.12–25.44)
Symptoms, signs and ill-defined conditions	33	0.79 (0.55–1.12)	170	1.08 (0.93–1.26)	118	0.84 (0.7–1.01)	77	0.84 (0.66–1.05)	398	0.92 (0.84–1.02)
Accidents and adverse effects	84	0.67 (0.54–0.84)^b^	342	0.73 (0.65–0.81)^b^	334	0.82 (0.74–0.92)^b^	222	0.76 (0.67–0.87)^b^	982	0.76 (0.71–0.81)^b^
Suicide and self-inflicted injury	36	0.99 (0.7–1.37)	148	1.13 (0.96–1.33)	114	1.07 (0.88–1.29)	61	0.87 (0.67–1.12)	359	1.04 (0.94–1.16)
Homicide and legal intervention	2	0.36 (0.04–1.31)	8	0.44 (0.19–0.86)^b^	7	0.51 (0.21–1.06)	6	0.73 (0.27–1.59)	23	0.5 (0.32–0.75)^b^
Other cause of death	359	0.65 (0.59–0.72)^b^	1860	0.86 (0.82–0.9)^b^	1759	0.88 (0.84–0.92)^b^	1300	0.86 (0.82–0.91)^b^	5278	0.85 (0.83–0.87)^b^

^1^Number of cancer patients who died due to each cause of death.

^2^95% Confidence interval.

^b^P-value < 0.05.

### Causes of death for regional cutaneous melanoma

We analyzed the data of 21,079 patients who were diagnosed with regional cutaneous melanoma, among whom 10,637 deaths were reported. The majority of these deaths occurred one to five years after diagnosis (n = 4591; 43.16%), whereas a conformable number of deaths occurred within the first year of diagnosis (n = 1697; 15.9%) and five to ten years after diagnosis (n = 1977; 18.59%). A distinct shift in the pattern of CODs was observed compared to that in the localized melanoma cohort. For example, the index melanoma accounted for most deaths (n = 7462; 70.15%) in the regional group. Unlike the previous localized pattern, non-melanoma cancers accounted for a mere (n = 526; 4.95%) of the deaths, and non-cancer causes accounted for (2649; 24.90%) deaths only. Additionally, a statistically significant increase in the risk of death due to multiple non-cancer CODs was detected compared with the general US population. The most common non-cancer CODs were attributed to diseases of heart (n = 945; 8.88%, SMR = 1.49; 95% CI, 1.4–1.59). Other common causes of death in this group were cerebrovascular diseases (n = 222, SMR = 1.67; 95% CI, 1.46–1.91), Alzheimer’s disease (n = 129, SMR = 1.26; 95% CI, 1.05–1.5), chronic obstructive pulmonary disease (n = 122, SMR = 0.8; 95% CI, 0.67–0.96), Diabetes mellitus (n = 118, SMR = 1.77; 95% CI, 1.46–2.11), and suicide and self-inflicted harm (n = 57, SMR = 2.49; 95% CI, 1.89–3.32) (Table 3, Fig. 1).

**Table 3:** Standardized mortality ratios for each cause of death following regional melanoma diagnosis.

Cause of death	Timing of deaths after diagnosis									
	< 1 year		1 to < 5 years		5 to < 10 years		≥ 10 years		Total	
	Observed	SMR (95% CI)	Observed	SMR (95% CI)	Observed	SMR (95% CI)	Observed	SMR (95% CI)	Observed	SMR (95% CI)
All	1697	3.34 (3.19–3.51)^b^	4591	5.93 (5.76–6.11)^b^	1977	3.83 (3.67–4.01)^b^	2372	1.88 (1.8–1.96)^b^	10,637	3.48 (3.41–3.54)^b^
Melanoma	1227	6.16 (5.82–6.51)^b^	5068	15.88 (15.45–16.33)^b^	978	12.69 (11.91–13.51)^b^	189	11.37 (9.81–13.12)^b^	7462	12.19 (11.92–12.47)^b^
Non-melanoma cancers	32	0.47 (0.32–0.66)^b^	246	1.15 (1.01–1.3)^b^	152	0.98 (0.83–1.15)	96	0.98 (0.8–1.2)	526	0.98 (0.9–1.07)
Non-cancer	438	1.86 (1.69–2.04)^b^	1254	1.69 (1.59–1.78)^b^	602	1.11 (1.02–1.2)^b^	355	0.98 (0.88–1.09)	2649	1.41 (1.35–1.46)^b^
In situ, benign or unknown behavior neoplasm	3	1.51 (0.31–4.41)	6	0.96 (0.35–2.09)	5	1.09 (0.35–2.55)	2	0.67 (0.08–2.41)	16	1.01 (0.58–1.64)
Tuberculosis	0	0 (0–70.22)	1	6.43 (0.16–35.83)	0	0 (0–34.87)	0	0 (0–56.77)	1	2.64 (0.07–14.71)
Septicemia	8	1.92 (0.83–3.78)	27	2.05 (1.35–2.98)^b^	13	1.35 (0.72–2.3)	8	1.25 (0.54–2.47)	56	1.68 (1.27–2.18)^b^
Other infectious and parasitic diseases including HIV	3	1.22 (0.25–3.57)	9	1.14 (0.52–2.17)	5	0.88 (0.29–2.05)	2	0.59 (0.07–2.12)	19	0.98 (0.59–1.53)
Diabetes mellitus	16	1.91 (1.09–3.1)^b^	57	2.14 (1.62–2.77)^b^	31	1.61 (1.1–2.29)^b^	14	1.11 (0.61–1.86)	118	1.77 (1.46–2.11)^b^
Alzheimer’s (ICD-9 and 10 only)	20	1.72 (1.05–2.66)^b^	51	1.32 (0.99–1.74)	37	1.23 (0.87–1.7)	21	0.94 (0.58–1.43)	129	1.26 (1.05–1.5)^b^
Diseases of heart	170	2.05 (1.75–2.38)^b^	470	1.85 (1.69–2.03)^b^	189	1.05 (0.91–1.21)	116	0.99 (0.82–1.18)	945	1.49 (1.4–1.59)^b^
Hypertension without heart disease	5	1.59 (0.52–3.72)	19	1.84 (1.11–2.87)^b^	7	0.89 (0.36–1.83)	3	0.54 (0.11–1.57)	34	1.26 (0.87–1.76)
Cerebrovascular diseases	34	1.96 (1.36–2.74)^b^	108	2.04 (1.68–2.47)^b^	51	1.36 (1.01–1.79)^b^	29	1.15 (0.77–1.66)	222	1.67 (1.46–1.91)^b^
Atherosclerosis	3	2.59 (0.53–7.58)	7	2.25 (0.9–4.63)	3	1.55 (0.32–4.53)	4	3.61 (0.98–9.23)	17	2.32 (1.35–3.72)^b^
Aortic aneurysm and dissection	3	1.96 (0.4–5.73)	8	1.79 (0.77–3.53)	0	0 (0–1.26)	0	0 (0–2.11)	11	1.03 (0.51–1.85)
Other diseases of arteries, arterioles, capillaries	3	2.55 (0.53–7.47)	4	1.11 (0.3–2.83)	2	0.78 (0.09–2.81)	2	1.18 (0.14–4.27)	11	1.21 (0.61–2.17)
Pneumonia and influenza	15	1.98 (1.11–3.26)^b^	37	1.63 (1.15–2.25)^b^	18	1.15 (0.68–1.82)	13	1.29 (0.69–2.21)	83	1.48 (1.18–1.84)^b^
Chronic obstructive pulmonary disease and allied conditions	26	1.38 (0.9–2.02)	56	0.93 (0.7–1.21)	24	0.54 (0.35–0.81)^b^	16	0.55 (0.31–0.89)^b^	122	0.8 (0.67–0.96)^b^
Stomach and duodenal ulcers	0	0 (0–8.98)	2	1.63 (0.2–5.88)	0	0 (0–4.34)	0	0 (0–6.69)	2	0.66 (0.08–2.38)
Chronic liver disease and cirrhosis	10	3.05 (1.46–5.6)^b^	19	1.73 (1.04–2.71)^b^	7	0.86 (0.35–1.77)	3	0.57 (0.12–1.66)	39	1.41 (1–1.93)^b^
Nephritis, nephrotic syndrome and nephrosis	10	1.73 (0.83–3.18)	34	1.87 (1.29–2.61)^b^	16	1.21 (0.69–1.97)	14	1.63 (0.89–2.74)	74	1.62 (1.27–2.03)^b^
Complications of pregnancy, childbirth, puerperium	1	70.44 (1.78–392.46)^b^	1	21.39 (0.54–119.18)	1	29.63 (0.75–165.08)	0	0 (0–193.21)	3	26.36 (5.44–77.05)^b^
Congenital anomalies	0	0 (0–11.86)	1	1.01 (0.03–5.64)	0	0 (0–5.26)	0	0 (0–8.23)	1	0.41 (0.01–2.28)
Symptoms, signs and ill-defined conditions	1	0.28 (0.01–1.57)	21	1.87 (1.16–2.85)^b^	11	1.31 (0.66–2.35)	6	1.18 (0.43–2.56)	39	1.38 (0.98–1.89)
Accidents and adverse effects	13	1.28 (0.68–2.18)	50	1.49 (1.1–1.96)^b^	23	0.93 (0.59–1.39)	15	0.91 (0.51–1.5)	101	1.19 (0.97–1.44)
Suicide and self-inflicted injury	16	5.66 (3.24–9.2)^b^	22	2.38 (1.49–3.6)^b^	12	1.79 (0.93–3.13)	7	1.7 (0.68–3.51)	57	2.49 (1.89–3.23)^b^
Homicide and legal intervention	1	2.26 (0.06–12.61)	0	0 (0–2.67)	1	1.07 (0.03–5.98)	0	0 (0–7.04)	2	0.61 (0.07–2.2)
Other cause of death	77	1.65 (1.31–2.07)^b^	244	1.59 (1.4–1.8)^b^	146	1.24 (1.05–1.46)^b^	80	0.97 (0.77–1.21)	547	1.37 (1.26–1.49)^b^

^1^Number of cancer patients who died due to each cause of death.

^2^95% Confidence interval.

^b^P-value < 0.05.

### Causes of death for distant cutaneous melanoma

Our data included 7614 reported deaths among the distant melanoma group (SMR, 14.25; 95% CI, 13.93–14.57). In contrast to the local and regional cohorts, most deaths in this group occurred within the first year of diagnosis (n = 5043). The second-highest number of recorded deaths occurred during the first to fifth year following the diagnosis period (n = 1857). Only a small fraction of deaths were reported from 5 to 10 years (n = 405), and after ten years of diagnosis follow-up periods (n = 309), highlighting the aggressive course of distant melanoma. Foreseeably, melanoma accounted for 6874 (90.28%) deaths in this group, while other non-melanoma cancers accounted for 109 (1.43%) deaths only. Compared with the general population, patients diagnosed with distant melanoma had an increased risk of death from the majority of non-cancer CODs, reflecting a general deterioration of health in this vulnerable group. We investigated 658 (8.64%) deaths due to non-cancer causes (SMR = 2.37; 95% CI, 2.19–2.56); of which 214 deaths were attributed to diseases of heart (SMR = 2.28; 95% CI, 1.99–2.61), 64 deaths to cerebrovascular diseases (SMR = 3.34; 95% CI, 2.57–4.26), 39 deaths to chronic obstructive pulmonary disease (SMR = 1.73; 95% CI, 1.23–2.36), and 30 deaths to Alzheimer’s disease (SMR = 2.05; 95% CI, 1.38–2.93) (Table 4, Fig. 1).

**Table 4:** Standardized mortality ratios for each cause of death following distant melanoma diagnosis.

Cause of death	Timing of deaths after diagnosis									
	< 1 year		1 to < 5 years		5 to < 10 years		≥ 10 years		Total	
	Observed	SMR (95% CI)	Observed	SMR (95% CI)	Observed	SMR (95% CI)	Observed	SMR (95% CI)	Observed	SMR (95% CI)
All	5043	32.85 (31.95–33.77)^b^	1857	12.83 (12.25–13.42)^b^	405	5.03 (4.55–5.54)^b^	309	1.99 (1.77–2.22)^b^	7614	14.25 (13.93–14.57)^b^
Melanoma	4715	48.82 (47.43–50.23)^b^	1971	28.87 (27.61–30.18)^b^	149	18.98 (16.05–22.28)^b^	12	8.59 (4.44–15.01)^b^	6847	39.33 (38.4–40.27)^b^
Non-melanoma cancers	31	2.45 (1.66–3.47)^b^	48	1.34 (0.99–1.78)	18	0.87 (0.52–1.38)	12	1.08 (0.56–1.88)	109	1.36 (1.11–1.64)^b^
Non-cancer	297	6.86 (6.11–7.69)^b^	243	2.02 (1.78–2.29)^b^	74	1.04 (0.82–1.31)	44	1.02 (0.74–1.37)	658	2.37 (2.19–2.56)^b^
In situ, benign or unknown behavior neoplasm	2	5.44 (0.66–19.66)	1	0.98 (0.02–5.46)	0	0 (0–6.03)	0	0 (0–10.31)	3	1.27 (0.26–3.72)
Septicemia	7	9.02 (3.63–18.59)^b^	7	3.24 (1.3–6.67)^b^	1	0.78 (0.02–4.32)	0	0 (0–4.96)	15	3.02 (1.69–4.98)^b^
Other infectious and parasitic diseases including HIV	4	8.72 (2.38–22.32)^b^	6	4.69 (1.72–10.22)^b^	3	4.07 (0.84–11.89)	0	0 (0–9.68)	13	4.55 (2.42–7.78)^b^
Diabetes mellitus	11	6.99 (3.49–12.5)^b^	8	1.78 (0.77–3.51)	2	0.77 (0.09–2.77)	2	1.37 (0.17–4.95)	23	2.27 (1.44–3.41)^b^
Alzheimer’s (ICD-9 and 10 only)	5	2.37 (0.77–5.52)	11	1.84 (0.92–3.3)	6	1.58 (0.58–3.43)	8	2.91 (1.25–5.72)^b^	30	2.05 (1.38–2.93)^b^
Diseases of heart	92	6.09 (4.91–7.47)^b^	85	2.08 (1.66–2.57)^b^	25	1.05 (0.68–1.56)	12	0.85 (0.44–1.48)	214	2.28 (1.99–2.61)^b^
Hypertension without heart disease	11	18.72 (9.34–33.49)^b^	2	1.19 (0.14–4.29)	0	0 (0–3.53)	1	1.5 (0.04–8.34)	14	3.52 (1.92–5.9)^b^
Cerebrovascular diseases	26	8.42 (5.5–12.34)^b^	26	3.16 (2.06–4.63)^b^	6	1.23 (0.45–2.69)	6	1.99 (0.73–4.34)	64	3.34 (2.57–4.26)^b^
Atherosclerosis	1	5.21 (0.13–29)	0	0 (0–8.4)	0	0 (0–15.2)	0	0 (0–27.96)	1	0.99 (0.03–5.54)
Aortic aneurysm and dissection	1	3.61 (0.09–20.14)	3	4.19 (0.86–12.25)	0	0 (0–9.47)	0	0 (0–18.01)	4	2.52 (0.69–6.46)
Other diseases of arteries, arterioles, capillaries	3	14.06 (2.9–41.07)^b^	2	3.48 (0.42–12.56)	0	0 (0–10.88)	0	0 (0–18.36)	5	3.76 (1.22–8.78)^b^
Pneumonia and influenza	8	5.88 (2.54–11.58)^b^	7	1.99 (0.8–4.1)	2	0.97 (0.12–3.5)	3	2.5 (0.52–7.31)	20	2.46 (1.5–3.79)^b^
Chronic obstructive pulmonary disease and allied conditions	18	5.14 (3.05–8.13)^b^	14	1.42 (0.78–2.39)	4	0.68 (0.19–1.74)	3	0.89 (0.18–2.59)	39	1.73 (1.23–2.36)^b^
Stomach and duodenal ulcers	1	13.53 (0.34–75.39)	0	0 (0–18.66)	0	0 (0–33.04)	0	0 (0–57.66)	1	2.24 (0.06–12.46)
Chronic liver disease and cirrhosis	2	3.08 (0.37–11.11)	3	1.52 (0.31–4.45)	0	0 (0–3.45)	0	0 (0–6.63)	5	1.18 (0.38–2.75)
Nephritis, nephrotic syndrome and nephrosis	8	7.47 (3.23–14.72)^b^	2	0.68 (0.08–2.44)	1	0.56 (0.01–3.12)	0	0 (0–3.58)	11	1.61 (0.8–2.88)
Complications of pregnancy, childbirth, puerperium	1	562.83 (14.25–3135.91)^b^	0	0 (0–805.16)	0	0 (0–1587.87)	0	0 (0–3222.61)	1	101.77 (2.58–567.02)^b^
Symptoms, signs and ill-defined conditions	7	11.13 (4.48–22.93)^b^	4	2.38 (0.65–6.08)	2	1.92 (0.23–6.94)	0	0 (0–6.19)	13	3.29 (1.75–5.63)^b^
Accidents and adverse effects	8	4.22 (1.82–8.32)^b^	10	1.79 (0.86–3.29)	5	1.59 (0.52–3.72)	1	0.55 (0.01–3.06)	24	1.93 (1.24–2.87)^b^
Suicide and self-inflicted injury	3	5.57 (1.15–16.29)^b^	6	3.75 (1.38–8.17)^b^	1	1.2 (0.03–6.69)	0	0 (0–8.74)	10	2.95 (1.41–5.42)^b^
Homicide and legal intervention	1	12.83 (0.32–71.49)	0	0 (0–16.76)	0	0 (0–34.37)	0	0 (0–65.56)	1	2.17 (0.05–12.07)
Other cause of death	77	8.89 (7.02–11.11)^b^	46	1.84 (1.35–2.46)^b^	16	1.04 (0.59–1.69)	8	0.82 (0.35–1.61)	147	2.5 (2.11–2.94)^b^

^1^Number of cancer patients who died due to each cause of death.

^2^95% Confidence interval.

^b^P-value < 0.05.

## Discussion

In our study, we analyzed the data of 224,624 malignant cutaneous melanomas in the USA retrieved from the SEER database. In this cohort, 60,110 deaths were observed across four distinct follow-up periods. Cutaneous melanomas of the face/ear and scalp/shoulder contributed to the highest number of deaths (48.01% and 35.295%, respectively). These findings are in line with those of previously published literature. For instance, Claeson et al.^7^ found that melanomas of the face, neck, and scalp were associated with the highest mortality. This strengthens the earlier suggestion that the anatomical site of melanoma greatly affects its prognosis^8,9^. In addition to the possibility of it being concealed by the hair, scalp melanoma is also more likely to be amelanotic than other head and neck sites^10^. This fact highlights the importance of scanning the head, neck, and scalp areas in asymptomatic patients during skin examination.

While the highest number of deaths among patients with localized disease was due to non-cancer causes, the highest number of deaths among patients with regional and distant cutaneous melanomas was attributed to index cancer. These findings might be explained by the better survival rate of patients with localized disease and a less aggressive disease course, shifting mortality to more benign causes. Nevertheless, 2649 (24.9%) and 658 (8.64%) deaths were due to non-cancer causes in the regional and distant groups, respectively. These findings warrant further investigation of non-cancer causes of death, especially among patients with localized disease.

Other secondary cancers were responsible for 6345 (10.56% of all deaths) deaths among the three groups. In the localized disease group, 5710 (13.64% of localized group deaths) deaths were attributed to non-melanoma cancers. The percentage was lower in the two other groups with 4.94% (n = 526) and 1.43% (n = 109) in regional and distant diseases, respectively. While the incidence of cutaneous melanoma has been increasing, especially in North America and Europe, survival rates have improved over the past years^11,12^. With increased survivability, there is an increased likelihood of developing second primary malignancies (SPMs). It is well-established that patients with malignant melanomas have an increased risk of developing SPMs^13,14^. Zheng et al.^15^ have analyzed the data from the Swedish Cancer Registry to assess the overall survival of patients suffering from melanoma and SPMs. They concluded that patients with malignant melanomas and SPMs had lower survival rates. They also determined variable survivability based on the location of the SPM. This highlights the importance of cancer screening in cutaneous melanoma survivors.

Deaths due to cardiovascular disease were the highest among the non-cancer causes in all three groups, with 10,098 deaths (16.8%) reported deaths from heart diseases. Many of these deaths can be attributed to the cardiotoxic effects of chemotherapy. From 1800 to 1950, surgical excision was the mainstay of melanoma treatment^16^. Starting in the 1960s, the use of adjuvant chemotherapy in melanoma management using a derivative of mustard gas (melphalan) and an alkylating agent (dacarbazine) were introduced. Unfortunately, neither intervention demonstrated efficacy in managing the disease^17,18^. Nevertheless, in the 1990s, new FDA-approved agents (stimulatory cytokines) were made available which were shown to be effective in some patients. However, these agents were associated with increased toxicity^18,19^. In the 2000s, a great leap in precision medicine and molecular profiling occurred, leading to molecularly-targeted therapies and immune-checkpoint inhibitors that improved overall survival compared to conventional therapies^20,21^. Regardless, the fact remains that many of these agents have cardiotoxic side effects which restrict their long-term use in melanoma patients. For instance, interleukin-2 (IL-2) and interferon-alpha (IFN-a), which are produced by T-lymphocytes and leukocytes, respectively, have been used clinically in the recombinant form for cancer management, and both have been associated with cardiotoxicity^22^. Additionally, IL-2 has been also associated with hemodynamic instability due to increased vascular permeability (capillary leak syndrome), and peripheral or pulmonary edema^22–24^. Furthermore, IL-2 can cause non-infectious myocarditis with acute troponin level, abnormal ECG, and decreased left ventricular function but normal coronary angiography^25–27^. IFN is associated with cardiotoxicities as well, albeit uncommon. Previous studies have reported an association between IFN and arrhythmias, myocardial infarctions, cardiomyopathies, and pericarditis^28–32^. Furthermore, targeted therapies, such as BRAF/MEK inhibitors, have been associated with arterial hypertension, venous thromboembolism, pulmonary embolism, and ECG abnormalities such as QTc prolongation^33–37^. Immune checkpoint inhibitors such as programmed cell death protein-1 (PD-1) and cytotoxic T-lymphocyte-associated antigen 4 (CTL 4) have been associated with myocarditis, pericardial disease, arrythmias, heart failure, and acute myocardial infarction^38–42^. The significance of our findings, considering previous findings, can shed the light on the importance of multidisciplinary approach in the management of melanoma patients to identify patients at risk of cardiovascular disease and ensure patient safety while minimizing any unnecessary treatment disruption.

In our analysis, 2314 (3.85% of all deaths) deaths due to cerebrovascular diseases across the three groups. Cerebrovascular disease was responsible for 2028, 222, and 64 deaths in localized, regional, and distant diseases, respectively. Deaths occurring between 1 and 10 years after the diagnosis of localized melanoma account for 68.98% of all cerebrovascular-disease-associated deaths. Cerebrovascular diseases among patients with melanoma can be attributed to chemotherapy, specifically immune checkpoint inhibitors. A systematic review and meta-analysis by Giustozzi et al.^43^ demonstrated a pooled stroke rate of 1.7% among patients using immune checkpoint inhibitors. They also reported a fatal stroke rate of 1.9%. Moreover, malignant melanoma metastasis can trigger a “stroke-mimicking” event^44^.

Alzheimer’s was responsible for 1828 deaths (3.04%) in all three groups. The localized CM group had the highest percentage of deaths attributed to Alzheimer’s disease (n = 1669; 3.99%), while deaths attributed to Alzheimer’s disease in the regional and distant CM groups accounted for a mere 222 (2.08%) and 64 (0.84%) deaths, respectively. Studies have reported an inverse relationship between cancer and Alzheimer’s disease^45,46^. Ibler et al.^47^ described the inverse relationship between melanoma and non-melanoma skin cancer, in relation to Alzheimer’s disease. However, studies on the exact association between melanoma and Alzheimer’s remain rather scarce. Research in this area is encouraged given the increased mortality rates due to Alzheimer’s disease among patients with cutaneous melanoma, as highlighted in the current study.

A total 1026 (1.71%) deaths due to diabetes mellitus were observed. The index melanoma accounted for the highest percentage of deaths among localized melanoma with 885 (2.11 of deaths in the localized group) deaths, while only 118 (1.1% of deaths in the regional group) and 23 (0.3% of deaths in the distant group) deaths were observed in the regional and distant disease groups, respectively. Diabetes itself is possibly implicated in the aggressiveness of melanomas, potentially indicating a correlation between the two conditions^48,49^. In fact, a systematic review and meta-analysis carried out by Qi et al.^50^ cite diabetes as a probable risk factor for malignant melanomas. However, some conflicts on the relationship between diabetes and melanoma can be found in the literature that warrant future research^51^. The treatment of melanoma, specifically with immune checkpoint inhibitor and interferon alpha, has also been reported to induce type 1 diabetes^52,53^. Pneumonia and influenza were associated with 869 (1.44%) deaths in our cohort; 766 deaths in the localized, 83 deaths in the regional, and 20 deaths in the distant CM groups. Similar to previous causes of death, melanoma treatment may be the culprit, specifically monoclonal antibodies targeting programmed cell death-1 (PD-1). Several studies have reported immunotherapy-related pneumonitis and subsequent pneumonia or organizing pneumonia in patients with melanoma^54–56^.

### Limitations

Our study has some limitations, namely, the inherent bias brought about by its retrospective nature. Additionally, we were unable to analyze the patients’ comorbidities or their quality of life. Moreover, we were unable to examine diverse CODs that were recorded as “other causes of death” as well as the specifics of deaths attributed to “accidents and adverse effects”. Additionally, the SEER database does not report the detailed chemotherapy and immunotherapy regimens administered to patients; thus, we could not to study the exact adverse effects and deaths associated with different therapy protocols. Finally, we relied heavily on the SEER staging variable for disease classification because of its consistency and wide coverage, which is different from the clinically used AJCC staging criteria.

## Conclusion

Non-cancer deaths represent a significant portion of the mortality in patients with malignant cutaneous melanoma. Compared to the general population, CM patients with regional and distant disease stages have an increased risk of death from most of the reported causes, especially cardiovascular and cerebrovascular diseases.

## Data Availability

All analyzed data are provided within the manuscript and its online supplementary material. Public access to the SEER database is available through https://seer.cancer.gov/.

## Abbreviations

CM: Cutaneous melanoma
SEER: Surveillance, epidemiology, and end results
SMR: Standardized mortality ratio
ICD-10: International classification of disease-tenth revision
O/E: Observed-to-expected ratio

## References

[1] Holmes D (2014). The cancer that rises with the sun. Nature.

[2] Haridas P, McGovern JA, McElwain SDL, Simpson MJ (2017). Quantitative comparison of the spreading and invasion of radial growth phase and metastatic melanoma cells in a three-dimensional human skin equivalent model. PeerJ.

[3] Yeh I, Bastian BC (2021). Melanoma pathology: New approaches and classification. Br. J. Dermatol..

[4] Domingues B, Lopes JM, Soares P, Pópulo H (2018). Melanoma treatment in review. Immunotargets Ther..

[5] Zaorsky NG (2017). Causes of death among cancer patients. Ann. Oncol..

[6] Khorana AA, Francis CW, Culakova E, Kuderer NM, Lyman GH (2007). Thromboembolism is a leading cause of death in cancer patients receiving outpatient chemotherapy. J. Thromb. Haemost..

[7] Claeson M (2020). Clinicopathological factors associated with death from thin (≤ 1·00 mm) melanoma. Br. J. Dermatol..

[8] Weinstock MA (1988). Effect of BANS location on the prognosis of clinical stage I melanoma: New data and meta-analysis. Br. J. Dermatol..

[9] Xie C (2017). Impact of scalp location on survival in head and neck melanoma: A retrospective cohort study. J. Am. Acad. Dermatol..

[10] Xie C (2017). Scalp melanoma: Distinctive high risk clinical and histological features. Austral. J. Dermatol..

[11] Crocetti E (2015). Survival of patients with skin melanoma in Europe increases further: Results of the EUROCARE-5 study. Eur. J. Cancer.

[12] Lyth J (2015). Trends in cutaneous malignant melanoma in Sweden 1997–2011: Thinner tumours and improved survival among men. Br. J. Dermatol..

[13] Chen T (2015). Risk of next melanoma in patients with familial and sporadic melanoma by number of previous melanomas. JAMA Dermatol..

[14] Chen T (2014). Multiple primary (even in situ) melanomas in a patient pose significant risk to family members. Eur. J. Cancer.

[15] Zheng G (2021). Types of second primary cancer influence overall survival in cutaneous melanoma. BMC Cancer.

[16] Keung EZ, Gershenwald JE (2018). The eighth edition American Joint Committee on Cancer (AJCC) melanoma staging system: Implications for melanoma treatment and care. Expert Rev. Anticancer Therapy.

[17] Mihich E (1971). Single agents in cancer chemotherapy, by R. B. Livingston and S. K. Carter. IFI/Plenum, New York, 1970, $20.00. J. Surg. Oncol..

[18] Richards JM, Gale D, Mehta N, Lestingi T (1999). Combination of chemotherapy with interleukin-2 and interferon alfa for the treatment of metastatic melanoma. JCO.

[19] Atkins MB (1999). High-dose recombinant interleukin 2 therapy for patients with metastatic melanoma: Analysis of 270 patients treated between 1985 and 1993. JCO.

[20] Lee C, Collichio F, Ollila D, Moschos S (2013). Historical review of melanoma treatment and outcomes. Clin. Dermatol..

[21] Pasquali S, Hadjinicolaou AV, Chiarion-Sileni V, Rossi CR, Mocellin S (2018). Systemic treatments for metastatic cutaneous melanoma. Cochrane Database Syst. Rev..

[22] Schechter D, Nagler A (1992). Recombinant interleukin-2 and recombinant interferon α immunotherapy cardiovascular toxicity. Am. Heart J..

[23] Gaynor ER (1988). The hemodynamic effects of treatment with interleukin-2 and lymphokine-activated killer cells. Ann. Intern. Med..

[24] Lee RE (1989). Cardiorespiratory effects of immunotherapy with interleukin-2. JCO.

[25] Eisner RM, Husain A, Clark JI (2004). Case report and brief review: IL-2 induced myocarditis. Cancer Invest..

[26] Thavendiranathan P, Verhaert D, Kendra KL, Raman SV (2011). Fulminant myocarditis owing to high-dose interleukin-2 therapy for metastatic melanoma. BJR.

[27] Kragel AH, Travis WD, Steis RG, Rosenberg SA, Roberts WC (1990). Myocarditis or acute myocardial infarction associated with interleukin-2 therapy for cancer. Cancer.

[28] Khakoo AY (2005). Reversible cardiomyopathy caused by administration of interferon α. Nat. Rev. Cardiol..

[29] Mocellin S, Pasquali S, Rossi CR, Nitti D (2010). Interferon alpha adjuvant therapy in patients with high-risk melanoma: A systematic review and meta-analysis. JNCI J. Natl. Cancer Inst..

[30] Nishio K, Arase T, Tada H, Tachibana H (2017). Interferon related pericarditis: Review. WJC.

[31] Ashraf F, Marmoush F, Shafi MI, Shah A (2016). Recurrent pericarditis, an unexpected effect of adjuvant interferon chemotherapy for malignant melanoma. Case Rep. Cardiol..

[32] Sleijfer S, Bannink M, Gool AR, Kruit WHJ, Stoter G (2005). side effects of interferon-α therapy. Pharm. World Sci..

[33] Larkin J (2014). Vemurafenib in patients with BRAF(V600) mutated metastatic melanoma: An open-label, multicentre, safety study. Lancet Oncol..

[34] Ascierto PA (2016). Cobimetinib combined with vemurafenib in advanced BRAF(V600)-mutant melanoma (coBRIM): Updated efficacy results from a randomised, double-blind, phase 3 trial. Lancet Oncol..

[35] Berger M (2020). Left ventricular ejection fraction decrease related to BRAF and/or MEK inhibitors in metastatic melanoma patients: A retrospective analysis. Cancer Med..

[36] Bronte E (2018). Cardiotoxicity mechanisms of the combination of BRAF-inhibitors and MEK-inhibitors. Pharmacol. Ther..

[37] Mincu RI (2019). Cardiovascular adverse events associated with BRAF and MEK inhibitors: A systematic review and meta-analysis. JAMA Netw. Open.

[38] D’Souza M (2021). The risk of cardiac events in patients receiving immune checkpoint inhibitors: A nationwide Danish study. Eur. Heart J..

[39] Moslehi JJ, Salem J-E, Sosman JA, Lebrun-Vignes B, Johnson DB (2018). Increased reporting of fatal immune checkpoint inhibitor-associated myocarditis. The Lancet.

[40] Salem J-E (2018). Cardiovascular toxicities associated with immune checkpoint inhibitors: An observational, retrospective, pharmacovigilance study. Lancet Oncol..

[41] Mahmood SS (2018). Myocarditis in patients treated with immune checkpoint inhibitors. J. Am. Coll. Cardiol..

[42] Lyon AR, Yousaf N, Battisti NML, Moslehi J, Larkin J (2018). Immune checkpoint inhibitors and cardiovascular toxicity. Lancet Oncol..

[43] Giustozzi M, Becattini C, Roila F, Agnelli G, Mandalà M (2021). Vascular events with immune checkpoint inhibitors in melanoma or non-small cell lung cancer: A systematic review and meta-analysis. Cancer Treat. Rev..

[44] Arakawa A (2020). A central nervous system metastasis of melanoma with stroke-like onset of left-lower quadrantanopsia. J. Gen. Fam. Med..

[45] Driver JA (2012). Inverse association between cancer and Alzheimer’s disease: Results from the Framingham Heart Study. BMJ.

[46] Roe CM (2010). Cancer linked to Alzheimer disease but not vascular dementia. Neurology.

[47] Ibler E (2018). Inverse association for diagnosis of Alzheimer’s disease subsequent to both melanoma and non-melanoma skin cancers in a large, urban, single-centre, Midwestern US patient population. J. Eur. Acad. Dermatol. Venereol..

[48] Kaneko A (2022). Relationship between type 2 diabetes mellitus and aggressiveness of melanoma. J. Dermatol. Sci..

[49] Straker RJ (2022). Association of type II diabetes mellitus with characteristics and outcomes for patients undergoing sentinel lymph node biopsy for cutaneous melanoma. J. Surg. Oncol..

[50] Qi L (2014). Type 2 diabetes mellitus and risk of malignant melanoma: A systematic review and meta-analysis of cohort studies. Iran. J. Public Health.

[51] Karlin NJ (2019). Survival and glycemic control in patients with coexisting melanoma and diabetes mellitus. Future Sci..

[52] Ng DB (2021). Pembrolizumab-induced type 1 diabetes in a 95-year-old veteran with metastatic melanoma. Fed. Pract..

[53] Chokr N, Farooq H, Guadalupe E (2018). Fulminant diabetes in a patient with advanced melanoma on nivolumab. Case Rep. Oncol. Med..

[54] Ma Q, Yang L, Gu F (2021). Immunotherapy-related pneumonitis and bacterial pneumonia after the successful treatment of metastatic malignant melanoma with pembrolizumab: A case report. Medicine.

[55] Hattori Y, Matsuyama K, Shu E, Seishima M (2019). Eosinophilic pneumonia and esophagitis in a patient with malignant melanoma treated with nivolumab. J. Dermatol..

[56] Sano T (2016). Nivolumab-induced organizing pneumonia in a melanoma patient. Jpn. J. Clin. Oncol..

